# Nephrologist Affiliation With Dialysis Facilities and Patient Trajectories in End-Stage Kidney Disease

**DOI:** 10.1001/jamanetworkopen.2026.6156

**Published:** 2026-04-16

**Authors:** Farbod Alinezhad, Gary J. Young, Richard Hirth, Jianfei Cao, Brady Post

**Affiliations:** 1 Department of Health Sciences, Bouve College of Health Sciences, Northeastern University, Boston, Massachusetts; 2 Center for Health Policy and Healthcare Research, Northeastern University, Boston, Massachusetts; 3 D’Amore-McKim School of Business, Northeastern University, Boston, Massachusetts; 4 Department of Health Management and Policy, University of Michigan School of Public Health, Ann Arbor; 5 Department of Internal Medicine, University of Michigan School of Medicine, Ann Arbor; 6 Institute for Healthcare Policy and Innovation, University of Michigan, Ann Arbor; 7 Department of Economics, College of Social Sciences and Humanities, Northeastern University, Boston, Massachusetts

## Abstract

**Importance:**

Across the treatment modalities for end-stage kidney disease (ESKD), kidney transplantation offers the best survival and quality-of-life outcomes, yet most patients are unable to receive a transplant, making dialysis the most common long-term treatment option. Despite its advantages, home peritoneal dialysis (PD) use remains limited in the US, with financial incentives potentially contributing to this underutilization.

**Objective:**

To examine the association between nephrologist affiliation with dialysis facilities and facility characteristics, including profit status and PD service offerings, with 2 critical ESKD outcomes: PD catheter placement and kidney transplant surgery.

**Design, Setting, and Participants:**

This retrospective cohort study was conducted using claims data from Medicare beneficiaries with ESKD who initiated dialysis between 2013 and 2022, with follow-up through 24 months after dialysis initiation. Data were analyzed from January 1, 2024, to December 31, 2025.

**Exposure:**

Nephrologist affiliation with dialysis facilities, determined by whether a nephrologist submitted any outpatient claims from a Medicare-certified dialysis facility.

**Main Outcomes and Measures:**

Outcomes included PD catheter placement and kidney transplant surgery within 24 months of dialysis initiation. Adjusted 24-month cumulative incidences and risk differences were estimated using a doubly robust survival targeted maximum likelihood estimation framework.

**Results:**

Among the 28 040 beneficiaries (mean [SD] age, 70.6 [10.8] years; 56.8% male) included in this study, 24 301 (86.7%) were attributed to affiliated nephrologists. The mean (SD) follow-up time was 322 (307) days. The adjusted 24-month cumulative incidence of PD catheter placement was 9.9% for patients of affiliated nephrologists compared with 8.2% for patients of nonaffiliated nephrologists (adjusted difference, 1.7 percentage points [pp] [95% CI, 0.6-2.8 pp]; *P* = .01). In stratified models, the adjusted difference in PD catheter placement was larger for nephrologists affiliated with for-profit facilities (1.9 pp [95% CI, 0.8-3.0 pp]; *P* = .01) and for those affiliated with facilities offering PD (2.0 pp [95% CI, 0.8-3.2 pp]; *P* = .01). Differences in kidney transplant surgery within 24 months were small and not statistically significant.

**Conclusions and Relevance:**

In this retrospective cohort study of Medicare beneficiaries initiating dialysis, nephrologist affiliation with dialysis facilities, especially for-profit centers or centers offering PD, was associated with a higher 24-month cumulative incidence of PD catheter placement, while the cumulative incidence of kidney transplant surgery within 24 months did not differ meaningfully by affiliation. These findings underscore the potential role of economic and organizational factors in promoting PD, and they highlight the need for policy reforms that expand home-based dialysis while preserving equitable access to transplantation.

## Key Points

**Question:**

Are nephrologist affiliations with dialysis facilities and facility characteristics associated with peritoneal dialysis (PD) catheter placement and kidney transplant surgery in patients initiating dialysis?

**Findings:**

In this cohort study of 28 040 Medicare beneficiaries initiating dialysis between 2013 and 2022 and followed up to 24 months, patients whose nephrologists were affiliated with a dialysis facility had a higher adjusted cumulative incidence of PD catheter placement than those treated by a nonaffiliated nephrologist. Adjusted differences in kidney transplant surgery within 24 months were small and not statistically significant; a negative control outcome (joint replacement surgery) showed no meaningful association with affiliation.

**Meaning:**

These findings suggest that although financial and institutional factors—including nephrologist affiliation with dialysis facilities—were associated with PD uptake, such affiliation did not limit early kidney transplant surgery.

## Teaser

This cohort study assesses whether nephrologist affiliation with dialysis facilities and facility characteristics are associated with peritoneal dialysis catheter placement and kidney transplant surgery in patients with end-stage kidney disease (ESKD) who initiate dialysis.

## Introduction

Multiple acceptable treatment modalities exist for end-stage kidney disease (ESKD). While kidney transplantation offers the best survival and quality-of-life outcomes with the lowest long-term costs, organ scarcity prevents many medically eligible patients from receiving a transplant.^1,2^ As a result, dialysis becomes either a bridge to transplantation or the primary long-term treatment for most patients. Within the currently available dialysis modalities, multiple sources, including clinical guidelines and health policy initiatives, identify home-based peritoneal dialysis (PD) as preferred to facility-based hemodialysis (HD) as the initial dialysis modality for most patients with ESKD who are eligible.^1,3,4,5,6^

Despite the benefits of PD and the steady increases in home dialysis over the last 15 years, the proportion of US patients receiving PD remains relatively low compared with many other countries.^1^ Barriers such as limited patient education, socioeconomic disparities, insufficient physician training, and health system–level financial incentive structures, particularly those embedded in payer reimbursement policies and dialysis facility payment models that historically favored in-center hemodialysis, may be implicated in this underutilization.^1,7,8,9,10^ Similarly, research indicates that financial incentives, including the ownership status of dialysis facilities, may influence multiple steps in transplant access pathways.^3,11,12,13,14^

The Centers for Medicare & Medicaid Services (CMS), the primary insurance provider for patients with ESKD in the US, has introduced successive payment reforms, from the 2011 prospective payment system to the End-Stage Renal Disease Treatment Choices (ETC) and Kidney Care Choices (KCC) models, to incentivize home dialysis and transplantation, with mixed results.^13,15,16,17,18^ These reforms primarily target dialysis organizations and nephrology practices through payment adjustments and shared savings–type incentives, and their impact plausibly depends on how financial and organizational incentives are translated to day-to-day clinical decisions, particularly during the transition from advanced chronic kidney disease to dialysis.

The economic features of dialysis treatment present a potentially important influence on how patients are treated. Specifically, in-center HD entails high fixed costs due to dedicated infrastructure, expensive equipment, and well-trained staff.^19^ These costs remain relatively constant regardless of the number of patients treated, so facilities typically need high patient volumes to spread out expenses and achieve profitability.^20^ By contrast, PD is generally performed at home and does not require as much upfront investment as in-center HD. On the one hand, in-center HD facilities, especially those operating on a for-profit model, may perceive PD or transplantation uptake as encroaching on their in-center patient pools. On the other hand, based on the aforementioned policy incentives from the CMS, some facilities might begin steering more patients toward PD, especially if they can simultaneously sustain a stable in-center HD patient pool. These facilities, in turn, might directly or indirectly influence the physicians they work with to change their modality education, referral, and preparation patterns accordingly.

Although some studies have examined the influence of dialysis facility and clinician factors on treatment modality decisions for patients with ESKD, the role of physician-facility relationships in shaping patient trajectories, particularly in the period leading up to dialysis initiation, remains unclear.^11,14,21,22^ The goals of this study were to (1) determine whether nephrologists’ affiliations with dialysis facilities is associated with the likelihood of PD catheter placement or kidney transplant surgery in patients with ESKD and (2) assess whether dialysis facility ownership type or types of services offered moderate the association between facility affiliation and PD catheter placement.

## Methods

This cohort study was deemed exempt from review by the Northeastern University Institutional Review Board because it involved secondary analysis of deidentified administrative claims data and did not constitute human participants research under the US Common Rule (45 CFR 46). The requirement for informed consent was waived because the study used existing deidentified data. This study followed the Strengthening the Reporting of Observational Studies in Epidemiology (STROBE) reporting guideline.

### Data Sources and Study Population

We used the 2013 to 2022 Medicare Administrative Claims Data for our analyses. Our dataset comprised a nationally representative 5% sample of all Medicare enrollees during these years. To collect demographic variables, monthly coverage indicators, and date of death, we used the Master Beneficiary Summary Files (rationale for using these data are provided in the eMethods in Supplement 1).

With the aforementioned datasets, we identified Medicare enrollees who started dialysis between 2013 and 2022. Briefly, we searched Medicare claims for dialysis service–related procedure codes, and we set the date of the first claim with such a procedure code as the date on which the patient started dialysis. We required patients to be enrolled in Medicare prior to their dialysis start date to enable a 1-year lookback for nephrologist attribution and baseline covariate measurement. We excluded patients residing outside the 50 US states and Washington, DC, and those with evidence of kidney transplantation prior to dialysis initiation (identified using both transplant surgery procedure codes and transplant status codes). Additionally, we excluded patients who originally became entitled to Medicare due to ESKD, as we could not ensure they did not start dialyzing prior to becoming enrolled. eTable 1 in Supplement 1 presents a list of the diagnosis and procedure codes we used.

### Nephrologist Identification and Affiliation

Nephrologists were identified as any physician listing nephrology as their primary or secondary specialty in the Medicare National Downloadable File.^23^ Affiliation was defined as submitting at least 1 outpatient claim from a Medicare-certified dialysis facility,^24^ and nephrologists were then classified by facility ownership type (for-profit only, nonprofit only, or both) and modality availability (HD only, HD plus PD, or mixed). Nephrologists classified as having mixed profit status or modality availability were those submitting claims across multiple dialysis facilities with differing ownership types, PD availability, or both, reflecting exposure to heterogeneous institutional environments and potentially competing financial incentives rather than a single, uniform incentive structure. Those with no facility claims were included in the not-affiliated reference group. To further characterize the practice setting, we also determined hospital employment and academic affiliation.^25^ Full details are presented in the eMethods in Supplement 1. Notably, being nonaffiliated does not imply that the nephrologist never sees patients receiving dialysis or is less engaged with their care; rather, it reflects the absence of claims from dedicated dialysis facilities and suggests that nephrology care is primarily delivered in alternative settings (eg, academic medical centers or outpatient clinics).

### Exposures

Each patient was assigned to the nephrologist from whom they received the plurality of their care, based on the highest number of distinct claims in the clinician file, during the 1-year period prior to their first dialysis. This predialysis year was chosen because it represents the critical phase during which patients receive nephrology care, such as modality education and preparatory management, that sets the trajectory for their subsequent dialysis treatment. Our primary exposures were as follows: (1) facility affiliation (whether the nephrologist was affiliated with any dialysis center); (2) ownership status of affiliated centers, classified as nonaffiliated, for-profit only (all affiliated centers were for-profit), nonprofit only (all affiliated centers were nonprofit), or mixed (some affiliated centers were for-profit and others were nonprofit); and (3) dialysis modalities offered at affiliated centers, classified as nonaffiliated, HD only (centers offering only hemodialysis), HD plus PD (centers offering both hemodialysis and peritoneal dialysis), or mixed (a combination of HD-only centers and HD plus PD centers). In the modality framework, *HD plus PD* refers to nephrologists whose affiliated facilities all offered both modalities, whereas *mixed* refers to nephrologists affiliated with some HD-only facilities and some HD plus PD facilities.

### Outcomes

We evaluated outcomes during the 24 months following dialysis initiation as follows: (1) PD catheter placement, determined by *Current Procedural Terminology* (*CPT*) codes 49418, 49421, and 49324; and (2) kidney transplant surgery, defined using the *International Classification of Diseases, Ninth Revision* (*ICD-9*) or *International Statistical Classification of Diseases and Related Health Problems* (*ICD-10*) procedure codes starting with 5569 and 0TY, respectively. We focused on PD catheter placement rather than PD use because there are no PD-specific *CPT* codes that reliably capture PD utilization, as available home dialysis billing codes do not distinguish PD from other home dialysis modalities. Because death is a nontrivial event in this population and transplant surgery can occur before PD catheter placement, our analyses of PD catheter placement treated death and kidney transplant surgery as competing events. We excluded PD catheter placements occurring more than 60 days before dialysis initiation (eMethods in Supplement 1).

We selected the 2-year follow-up period based on the typical timing of the main outcomes. Most of the patients included in our analysis received a PD catheter within about 1 year and a transplant within 24 months of dialysis initiation, making a 2-year window sufficient to capture both events reliably.

In addition, we evaluated the association between our exposure variables and the receipt of joint replacement or repair surgery as a negative control outcome.^26^ Since there is no plausible clinical link between nephrologist affiliation or facility characteristics and the likelihood of undergoing joint replacement surgery, any association would have indicated residual confounding.^26^

### Covariates

Baseline covariates included patient demographic variables (age, sex, race and ethnicity, and rural or urban residence as determined by Rural-Urban Continuum Codes), comorbid conditions, and community context variables (derived from US Census data).^27^ We also included the number of months during the predialysis year with full Medicare Part A and Part B coverage and dual eligibility for Medicare and Medicaid as covariates. In addition, we included the calendar year of the first outcome year as a covariate to account for temporal effects. Race and ethnicity were obtained from Medicare enrollment information in the Master Beneficiary Summary File and were analyzed using the categories available in the data (American Indian or Alaska Native, Asian, Black, Hispanic, or White). Race and ethnicity data were included because prior literature indicated that substantial differences in dialysis care trajectories and access to preferred kidney failure treatments may exist across racial and ethnic groups.^28^ We therefore considered these variables relevant both for confounding control and for assessment of heterogeneity. The eMethods in Supplement 1 includes a list of comorbidities^29^ and detailed covariate definitions.

### Statistical Analysis

To estimate adjusted differences in the 24-month cumulative incidence of each outcome across exposure groups, we used a doubly robust survival targeted maximum likelihood estimation (TMLE) approach implemented in R, version 4.3.3 (R Project for Statistical Computing). This method combines modeling of treatment assignment and censoring with modeling of the outcome hazard, providing robustness against misspecification if at least 1 of these model components is correctly specified.^30,31,32^ For PD catheter placement, we used a competing risks framework with death and kidney transplant surgery as competing events. For transplant surgery and joint replacement surgery, death was treated as a competing event (eMethods in Supplement 1).

Patients were followed up from dialysis initiation until either the earliest of 24 months, the administrative end of the dataset, or loss of fee-for-service Medicare observability (eMethods in Supplement 1). We reported adjusted 24-month cumulative incidence under each exposure level and risk differences (percentage-point [pp] differences) comparing each exposure level with the not-affiliated reference group, along with 95% CIs and 2-tailed *P* values. Statistical significance was assessed at an α = .05 level. Missing data handling procedures are described in the eMethods in Supplement 1. Because multiple comparisons were evaluated for each outcome across several affiliation specifications (eg, profit status and modality categories), Holm and Benjamini-Hochberg adjusted *P* values were computed within each outcome to account for multiple comparisons.^33,34^

We performed sensitivity analyses to assess robustness to alternate specifications: (1) restricting to nephrologists with more precise affiliation patterns (≥75% of outpatient claims consistent with the assigned category), (2) restricting to dialysis initiations through 2019 to minimize potential effects of the COVID-19 era and later market changes, and (3) restricting to patients whose first observed nephrology encounter occurred at least 30 days before dialysis initiation (no crash starts), to allow sufficient time for the nephrologist relationship to affect treatment trajectories.

We also conducted prespecified subgroup analyses by race and ethnicity, geographic location (rurality), and sex to assess potential heterogeneity of associations. Data were analyzed from January 1, 2024, to December 31, 2025.

## Results

### Patient Characteristics and Unadjusted Outcomes

Our main analytic sample included 28 040 patients receiving dialysis, of whom 24 301 (86.7%) had an affiliated nephrologist and 3739 (13.3%) had a nonaffiliated nephrologist. Their mean (SD) age was 70.6 (10.8) years; 56.8% were male and 43.2% were female. In terms of race and ethnicity, 1.0% of patients were American Indian or Alaska Native, 2.7% were Asian, 21.0% were Black, 3.5% were Hispanic, and 68.7% were White. The mean (SD) follow-up time was 322 (307) days. The eResults in Supplement 1 provides details on inclusion criteria, eFigure 1 in Supplement 1 provides the sample flow diagram, and eTable 2 in Supplement 1 provides follow-up time statistics. Baseline characteristics were generally similar across groups, with modest differences in rural residence and county-level socioeconomic indicators (Table and eTable 3 in Supplement 1).

**Table.** Baseline Demographic and Clinical Characteristics of Medicare Beneficiaries Initiating Dialysis by Nephrologist Affiliation and Facility Profit Status^a^

Characteristic	Overall patients (N = 27 678)	Nephrologist dialysis facility affiliation			
		Nonaffiliated (n = 3883)	Affiliated with both profit statuses (n = 3797)	Affiliated with for-profit facilities (n = 17 354)	Affiliated with nonprofit facilities (n = 2644)
**Demographic**					
Age, mean (SD), y	70.6 (10.8)	69.8 (11.2)	70.9 (10.7)	70.9 (10.7)	69.7 (10.9)
Sex					
Female	11 940 (43.1)	1674 (43.1)	1633 (43.0)	7532 (43.4)	1100 (41.6)
Male	15 738 (56.9)	2209 (56.9)	2164 (57.0)	9822 (56.6)	1544 (58.4)
Race and ethnicity					
American Indian or Alaska Native	263 (1.0)	39 (1.0)	48 (1.3)	141 (0.8)	37 (1.4)
Asian	744 (2.7)	93 (2.4)	100 (2.6)	491 (2.8)	60 (2.3)
Black	5815 (21.0)	817 (21.0)	781 (20.6)	3729 (21.5)	488 (18.5)
Hispanic	955 (3.5)	119 (3.1)	130 (3.4)	632 (3.6)	75 (2.8)
White	19 020 (68.7)	2682 (69.1)	2618 (69.0)	11 814 (68.1)	1906 (72.1)
Missing	881 (3.2)	133 (3.4)	120 (3.2)	547 (3.2)	78 (3.0)
Rural residence	5177 (18.7)	637 (16.4)	707 (18.6)	3248 (18.7)	584 (22.1)
County-level context, mean (SD)					
County population, per 100 000	10.0 (18.4)	11.4 (20.2)	7.5 (11.9)	10.6 (19.8)	7.6 (11.5)
Median household income, $1000s	59.7 (17.9)	62.4 (18.9)	59.2 (18.0)	59.0 (17.6)	60.7 (18.0)
Poverty rate, %^b^	15.0 (5.6)	14.6 (5.6)	15.0 (5.6)	15.2 (5.6)	14.3 (5.4)
Unemployment rate, %	7.6 (2.7)	7.0 (2.6)	7.5 (2.8)	7.3 (2.7)	7.1 (2.8)
High school or higher educational attainment, %	49.6 (8.4)	48.3 (8.8)	50.0 (8.5)	49.8 (8.3)	49.2 (8.9)
**Clinical**					
Comorbidities					
Myocardial infarction	6923 (25.0)	991 (25.5)	942 (24.8)	4326 (24.9)	663 (25.1)
Congestive heart failure	16 507 (59.6)	2238 (57.6)	2266 (59.7)	10 464 (60.3)	1542 (58.3)
Peripheral vascular disease	10 043 (36.3)	1397 (36.0)	1429 (37.6)	6270 (36.1)	946 (35.8)
Cerebrovascular disease	8001 (28.9)	1112 (28.6)	1077 (28.4)	5110 (29.4)	707 (26.7)
Dementia	1907 (6.9)	252 (6.5)	267 (7.0)	1243 (7.2)	145 (5.5)
Chronic pulmonary disease	10 937 (39.5)	1511 (38.9)	1514 (39.9)	6925 (39.9)	993 (37.6)
Rheumatoid disease	1672 (6.0)	261 (6.7)	222 (5.8)	1038 (6.0)	151 (5.7)
Peptic ulcer disease	1384 (5.0)	210 (5.4)	176 (4.6)	861 (5.0)	137 (5.2)
Mild liver disease	3005 (10.9)	468 (12.1)	423 (11.1)	1826 (10.5)	290 (11.0)
Diabetes	4240 (15.3)	600 (15.5)	633 (16.7)	2568 (14.8)	439 (16.6)
Diabetes with complications	14 542 (52.5)	1886 (48.6)	1999 (52.6)	9305 (53.6)	1352 (51.1)
Hemiplegia or paraplegia	894 (3.2)	138 (3.6)	107 (2.8)	580 (3.3)	70 (2.6)
Nonmetastatic cancer	4448 (16.1)	677 (17.4)	597 (15.7)	2712 (15.6)	463 (17.5)
Metastatic cancer	1010 (3.6)	167 (4.3)	127 (3.3)	611 (3.5)	105 (4.0)
Moderate to severe liver disease	1270 (4.6)	277 (7.1)	145 (3.8)	703 (4.1)	146 (5.5)
HIV/AIDS	224 (0.8)	35 (0.9)	29 (0.8)	128 (0.7)	32 (1.2)
Mortality rate	4478 (16.2)	696 (17.9)	589 (15.5)	2761 (15.9)	433 (16.4)
No. of hospitalized days, mean (SD)	11.3 (18.8)	12.2 (19.8)	10.7 (17.6)	11.3 (19.0)	10.4 (17.9)
Outcomes					
Peritoneal dialysis catheter placement	2219 (8.0)	257 (6.6)	307 (8.1)	1473 (8.5)	182 (6.9)
Kidney transplantation	335 (1.2)	61 (1.6)	46 (1.2)	182 (1.1)	44 (1.7)
Joint replacement surgery	836 (3.0)	104 (2.7)	120 (3.2)	522 (3.0)	89 (3.4)

^a^ Unless specified otherwise, values are presented as the No. (%) of patients. The difference in the total number of patients herein and in eTable 1 in Supplement 1 arises from additional missing data on nephrologist profit status.

^b^ Defined as the percentage of county residents whose family income in the past 12 months was below the Census Bureau’s official poverty threshold for their family size and composition, based on American Community Survey 5-year estimates.

The unadjusted 24-month percentage of PD catheter placement was higher among patients of affiliated nephrologists (8.3%) compared with patients of nonaffiliated nephrologists (6.5%). The unadjusted percentage of kidney transplant surgery was 1.6% among patients of nonaffiliated nephrologists and 1.2% among patients of affiliated nephrologists (eTables 3 and 4 in Supplement 1). When stratified by facility ownership, PD catheter placement was highest among patients of nephrologists affiliated with for-profit facilities (8.5%) (Table).

### Adjusted Results

The Figure shows the results of our survival TMLE models and includes the point estimates and 95% CIs corresponding to the plotted comparisons. The adjusted 24-month cumulative incidence of PD catheter placement was 9.9% for patients of affiliated nephrologists compared with 8.2% for patients of nonaffiliated nephrologists, yielding an adjusted difference of 1.7 pp (95% CI, 0.6-2.8 pp; Holm-adjusted *P* = .01).

**Figure.**
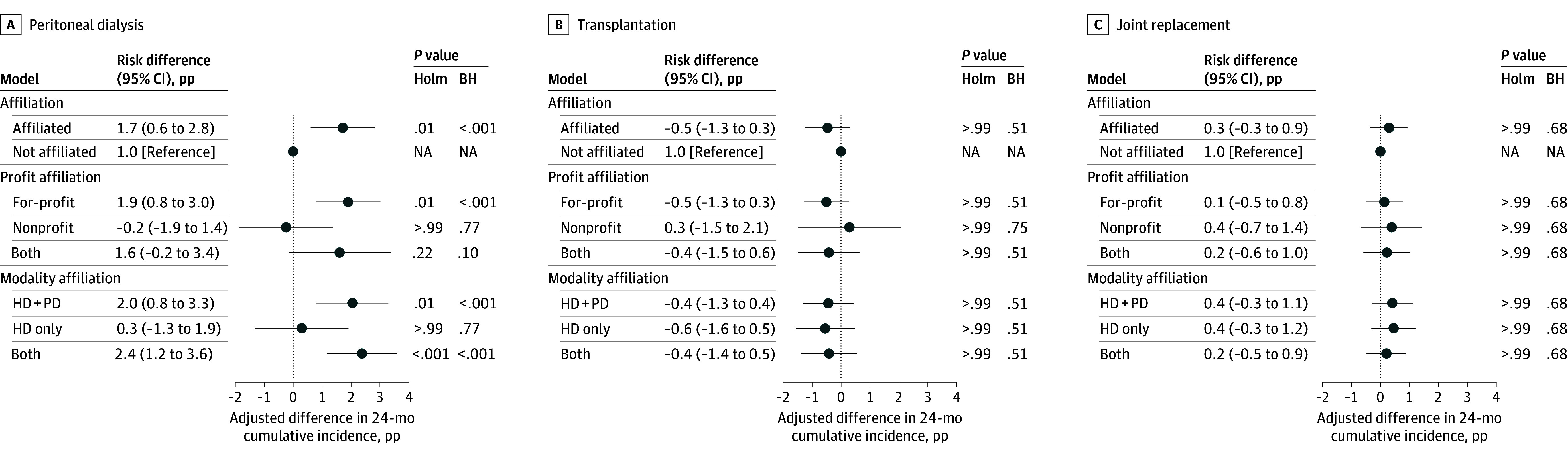
Forest Plots of Adjusted Differences in Peritoneal Dialysis (PD) Catheter Placement, Kidney Transplant Surgery, and Joint Replacement Surgery by Nephrologist Affiliation, Profit Status, and Dialysis Modalities Offered Points indicate adjusted 24-month risk differences; horizontal lines indicate 95% CIs. Holm and Benjamini-Hochberg (BH) multiplicity-adjusted *P* values are reported for each outcome. HD indicates hemodialysis; HD+PD, affiliated facilities all offered both HD and PD modalities; NA, not applicable; pp, percentage points.

When stratified by ownership, the higher adjusted 24-month incidence of PD catheter placement among affiliated nephrologists was observed primarily for for-profit affiliations (adjusted difference, 1.9 pp [95% CI, 0.8-3.0 pp]; Holm-adjusted *P* = .01); estimates for nonprofit-only and mixed affiliations were smaller and not clearly distinguishable from the nonaffiliated group. When stratified by modality availability, PD catheter placement was higher among nephrologists affiliated with facilities offering PD (HD plus PD and mixed-modality affiliations) (adjusted difference, 2.0 pp [95% CI, 0.8-3.2 pp]; Holm-adjusted *P* = .01), whereas nephrologists with HD-only affiliations showed little difference from nonaffiliated nephrologists. In contrast, kidney transplant surgery within 24 months and the negative control outcome (joint replacement surgery) showed no meaningful differences by affiliation, including across ownership and modality strata.

### Sensitivity and Subgroup Analyses

Sensitivity analyses showed directionally similar results for PD catheter placement and similarly null findings for transplant surgery, with greater variability in estimates reflecting smaller effective sample sizes (eFigures 2-4 in Supplement 1). Subgroup analyses indicated some heterogeneity, with more precise associations in urban, male, and White subgroups and less precise estimates in rural and racial and ethnic minority groups, while overall trends were generally similar across most subgroups (eFigures 5-7 in Supplement 1).

## Discussion

In this retrospective cohort analysis of patients receiving incident dialysis, we examined the association between nephrologist dialysis facility affiliation and the likelihood of PD catheter placement and kidney transplant surgery within 24 months of dialysis initiation. Our findings suggest that patients of dialysis facility-affiliated nephrologists are more likely to receive PD catheter placement compared with patients of nonaffiliated neurologists. Furthermore, the difference in the adjusted 24-month cumulative incidence of receiving a PD catheter was primarily observed when nephrologists were affiliated with for-profit facilities, facilities that offered PD, or both. We did not observe meaningful differences in kidney transplant surgery cumulative incidence within 24 months across the groups.

Although the absolute differences we observed were modest (on the order of 1.5-2.5 pp), the relative differences can be meaningful in the context of low baseline PD uptake. For example, a 1.7-pp absolute increase corresponds to an approximately 20% relative increase compared with an adjusted cumulative incidence of 8.2% in the nonaffiliated group, suggesting that organizational alignment may matter even when overall PD use remains low.

To our knowledge, no previous studies have specifically compared nonaffiliated nephrologists with affiliated ones; however, Lin et al^21^ found that nephrologist-owned dialysis facilities had higher home dialysis use. While we did not focus on nephrologist ownership of dialysis facilities, our findings are consistent with the broader notion that stronger physician-facility alignment, whether via ownership or other forms of affiliations, may foster an environment supportive of home-based therapies. In particular, nephrologists affiliated with for-profit facilities or facilities offering PD may devote additional resources, such as staffing, patient education, or specialized PD training, in response to financial and organizational advantages tied to home dialysis. They may also benefit from implicit incentives, including streamlined workflows and scheduling priorities that facilitate smoother transitions to PD. Because PD remains underutilized in the US, facilities offering both HD and PD likely have a sufficient in-center HD patient base to fully utilize their in-center resources; as a result, they may be more inclined to expand PD, which entails lower fixed costs and can be more profitable under current reimbursement structures.^1,20^

In contrast, our findings do not suggest that predialysis nephrologist-facility affiliation was associated with lower receipt of kidney transplant surgery in the first 24 months after dialysis initiation. We assessed transplant surgery rather than earlier steps such as referral, evaluation, or waitlisting, and these outcomes should not be conflated. Differences between our findings and prior work focused on facility-level waiting list metrics may reflect both the outcome definition and the fact that we focused on predialysis physician practice patterns, whereas facility-level analyses often reflect postinitiation care processes and organizational policies.^11,12,13,14^

Although existing Medicare initiatives such as ETC and KCC seek to incentivize home-based dialysis, the persistently low rates of PD in the US suggest that current strategies have yet to achieve their full potential.^1,17,18^ Our findings are consistent with the possibility that organizational environments that include PD capability and that are positioned to respond to incentive programs may be associated with higher PD uptake. Further policy measures, such as clearer alignment of facility- and physician-level incentives, standardized PD education expectations, and support for PD training and infrastructure, could help narrow this gap. In addition, given substantial patient-level and community-level barriers to PD access, aligning incentives alone is unlikely to be sufficient without parallel efforts to address socioeconomic, educational, and structural barriers.^7^

### Limitations

This study has some limitations. Similar to many other studies using administrative claims data, our study may have limited granularity. Certain unobserved patient-level factors, such as home environment suitability for PD, caregiver support, or patient preferences, could remain confounders. Moreover, claims-based exposure definitions may misattribute patients who saw multiple nephrologists in the predialysis year; although we used plurality-claims attribution and conducted sensitivity analyses, misclassification could attenuate associations.

Second, follow-up time was often shorter than 24 months due to administrative end-of-data truncation for later dialysis initiators and censoring related to Medicare coverage changes. We addressed this by using a survival TMLE framework with explicit censoring and competing risks, but residual bias could remain if censoring is informative beyond measured covariates.

Third, facility-level contextual factors (eg, chain affiliation, staffing ratios, or PD training programs) are not directly measured in claims. These organizational characteristics may be part of the mechanisms through which affiliation operates. We view such contextual differences as potential mediators of the observed associations and as an inherent part of why certain facilities, and the physicians affiliated with them, may behave differently. While a formal mediation analysis was beyond the scope of our study, examining these pathways represents an important direction for future research.

Fourth, despite using a doubly robust estimation approach to mitigate measured confounding, residual unmeasured confounding cannot be ruled out. Accordingly, our findings should be interpreted as associations rather than definitive causal effects.

Finally, because we excluded beneficiaries with ESKD-based Medicare entitlement, our cohort was older than the full incident ESKD population. This may limit generalizability to younger patients and to populations whose predialysis nephrology care is not captured in Medicare claims.

## Conclusions

In this retrospective cohort study of Medicare beneficiaries initiating dialysis between 2013 and 2022, nephrologist affiliation with a dialysis facility, particularly those under for-profit ownership or offering PD, was associated with a higher adjusted 24-month cumulative incidence of PD catheter placement, whereas kidney transplant surgery within 24 months appeared largely unaffected by such affiliation arrangements. Given the persistently low utilization of PD in the US, there is an opportunity for policy interventions that leverage these existing incentives and organizational structures to further expand PD, while ensuring equitable access to transplantation and other preferred kidney failure treatments.

## Notes

Supplement 1.
eMethods

eResults
**eTable 1.** Diagnosis Codes and Healthcare Common Procedure Coding System (HCPCS) Codes for Identifying Patients With End-Stage Kidney Disease, Dialysis Procedures, and Kidney Transplantation **eFigure 1.** Sample Flow Diagram **eTable 2.** Follow-Up Time, Outcome Events, and Censoring in the Analytic Cohort **eTable 3.** Baseline Demographic and Clinical Characteristics of Medicare Beneficiaries Initiating Dialysis **eTable 4.** Baseline Demographic and Clinical Characteristics of Medicare Beneficiaries Initiating Dialysis by Nephrologist Affiliation Status and Dialysis Modalities Offered at the Place of Affiliation **eFigure 2.** Adjusted Differences in Peritoneal Dialysis Catheter Placement, Kidney Transplant Surgery, and Joint Replacement Surgery by Nephrologist Affiliation, Profit Status, and Dialysis Modalities Offered (Strong Affiliation ≥75%) **eFigure 3.** Adjusted Differences in Peritoneal Dialysis Catheter Placement, Kidney Transplant Surgery, and Joint Replacement Surgery by Nephrologist Affiliation, Profit Status, and Dialysis Modalities Offered (Dialysis Initiation Through 2019; Pre–COVID-19 Era) **eFigure 4.** Adjusted Differences in Peritoneal Dialysis Catheter Placement, Kidney Transplant Surgery, and Joint Replacement Surgery by Nephrologist Affiliation, Profit Status, and Dialysis Modalities Offered (No Crash Starts) **eFigure 5.** Sex-Stratified Adjusted Differences in Peritoneal Dialysis Catheter Placement by Nephrologist Affiliation, Profit Status, and Dialysis Modalities Offered **eFigure 6.** Race and Ethnicity–Stratified Adjusted Differences in Peritoneal Dialysis Catheter Placement by Nephrologist Affiliation, Profit Status, and Dialysis Modalities Offered **eFigure 7.** Rurality-Stratified Adjusted Differences in Peritoneal Dialysis Catheter Placement by Nephrologist Affiliation, Profit Status, and Dialysis Modalities Offered

Supplement 2.
Data Sharing Statement
