# Supplementary material for: Nephrologist Affiliation With Dialysis Facilities and Patient Trajectories in End-Stage Kidney Disease
Source: JAMA Netw Open. 2026 Apr 16;9(4):e266156. doi: 10.1001/jamanetworkopen.2026.6156 (PMC13087812; doi:10.1001/jamanetworkopen.2026.6156)
Supplement: Supplement 1. — eMethods eResults eTable 1. Diagnosis Codes and Healthcare Common Procedure Coding System (HCPCS) Codes for Identifying Patients With End-Stage Kidney Disease, Dialysis Procedures, and Kidney Transplantation eFigure 1. Sample Flow Diagram eTable 2. Follow-Up Time, Outcome Events, and Censoring in the Analytic Cohort eTable 3. Baseline Demographic and Clinical Characteristics of Medicare Beneficiaries Initiating Dialysis eTable 4. Baseline Demographic and Clinical Characteristics of Medicare Beneficiaries Initiating Dialysis by Nephrologist Affiliation Status and Dialysis Modalities Offered at the Place of Affiliation eFigure 2. Adjusted Differences in Peritoneal Dialysis Catheter Placement, Kidney Transplant Surgery, and Joint Replacement Surgery by Nephrologist Affiliation, Profit Status, and Dialysis Modalities Offered (Strong Affiliation ≥75%) eFigure 3. Adjusted Differences in Peritoneal Dialysis Catheter Placement, Kidney Transplant Surgery, and Joint Replacement Surgery by Nephrologist Affiliation, Profit Status, and Dialysis Modalities Offered (Dialysis Initiation Through 2019; Pre–COVID-19 Era) eFigure 4. Adjusted Differences in Peritoneal Dialysis Catheter Placement, Kidney Transplant Surgery, and Joint Replacement Surgery by Nephrologist Affiliation, Profit Status, and Dialysis Modalities Offered (No Crash Starts) eFigure 5. Sex-Stratified Adjusted Differences in Peritoneal Dialysis Catheter Placement by Nephrologist Affiliation, Profit Status, and Dialysis Modalities Offered eFigure 6. Race and Ethnicity–Stratified Adjusted Differences in Peritoneal Dialysis Catheter Placement by Nephrologist Affiliation, Profit Status, and Dialysis Modalities Offered eFigure 7. Rurality-Stratified Adjusted Differences in Peritoneal Dialysis Catheter Placement by Nephrologist Affiliation, Profit Status, and Dialysis Modalities Offered [file jamanetwopen-e266156-s001.pdf]

Supplementary Online Content

Alinezhad F, Young GJ, Hirth R, Cao J, Post B. Nephrologist affiliation with dialysis facilities and patient trajectories in end-stage kidney disease. *JAMA Netw Open*. 2026;9(4):e266156. doi:10.1001/jamanetworkopen.2026.6156

eMethods
eResults
eTable 1. Diagnosis Codes and Healthcare Common Procedure Coding System (HCPCS) Codes for Identifying Patients With End-Stage Kidney Disease, Dialysis Procedures, and Kidney Transplantation
eFigure 1. Sample Flow Diagram
eTable 2. Follow-Up Time, Outcome Events, and Censoring in the Analytic Cohort
eTable 3. Baseline Demographic and Clinical Characteristics of Medicare Beneficiaries Initiating Dialysis
eTable 4. Baseline Demographic and Clinical Characteristics of Medicare Beneficiaries Initiating Dialysis by Nephrologist Affiliation Status and Dialysis Modalities Offered at the Place of Affiliation
eFigure 2. Adjusted Differences in Peritoneal Dialysis Catheter Placement, Kidney Transplant Surgery, and Joint Replacement Surgery by Nephrologist Affiliation, Profit Status, and Dialysis Modalities Offered (Strong Affiliation ≥75%)
eFigure 3. Adjusted Differences in Peritoneal Dialysis Catheter Placement, Kidney Transplant Surgery, and Joint Replacement Surgery by Nephrologist Affiliation, Profit Status, and Dialysis Modalities Offered (Dialysis Initiation Through 2019; Pre–COVID-19 Era)
eFigure 4. Adjusted Differences in Peritoneal Dialysis Catheter Placement, Kidney Transplant Surgery, and Joint Replacement Surgery by Nephrologist Affiliation, Profit Status, and Dialysis Modalities Offered (No Crash Starts)
eFigure 5. Sex-Stratified Adjusted Differences in Peritoneal Dialysis Catheter Placement by Nephrologist Affiliation, Profit Status, and Dialysis Modalities Offered
eFigure 6. Race and Ethnicity–Stratified Adjusted Differences in Peritoneal Dialysis Catheter Placement by Nephrologist Affiliation, Profit Status, and Dialysis Modalities Offered
eFigure 7. Rurality-Stratified Adjusted Differences in Peritoneal Dialysis Catheter Placement by Nephrologist Affiliation, Profit Status, and Dialysis Modalities Offered

This supplementary material has been provided by the authors to give readers additional information about their work.

© 2026 Alinezhad et al. *JAMA Network Open*.

eMethods

Data Sources Selection Rationale

We used Medicare claims rather than United States Renal Data System (USRDS) because our primary exposure is defined at the nephrologist level using outpatient billing patterns across settings in the year prior to dialysis initiation (including non-dialysis care), which requires complete pre-dialysis physician claims. Our analytic cohort also excluded beneficiaries with ESRD-based Medicare entitlement at dialysis initiation to ensure a full pre-dialysis Medicare observation window.

Missing Data Handling Procedures

To address missing data for nephrologist affiliation and dialysis facility characteristics, we implemented a combined imputation strategy using both last observation carried forward (LOCF) and backward imputation, with a maximum allowable gap of 6 months. In the LOCF approach, if a variable was missing at a given time point, we imputed the value using the most recent non-missing observation that occurred before the missing point (within a 6-month window). Conversely, if data were missing at the beginning of the exposure period (i.e., no prior observation was available), we applied backward imputation. This involved identifying the first available observation after the missing period (within a 6-month window) and carrying that value backward to impute the missing data. Initially, among the 28,738 unique patients who met our inclusion criteria, 2,719 were assigned to a nephrologist with a missing affiliation status during the exposure period. By applying these imputation methods, we reduced the number of patients with missing affiliation status for their attributed nephrologist to 547. We had little to no missingness for the patient-related covariates.

Exposures

We determined the exposure variables for each patient's nephrologist using claims data from the one-year period ending on the first day of the month in which the patient-initiated dialysis (to avoid post-baseline leakage).

Since only a very small percentage of nephrologists were affiliated with facilities that only offered peritoneal dialysis, this group was not included in our analyses. Dialysis facility ownership status and modality characteristics were obtained from the Dialysis Facility Report (DFR), which draws on CMS administrative facility data reported through the End-Stage Renal Disease Quality Reporting System (EQRS).

Comorbidities

Our exposure year covariates included patient demographic variables, including age, sex, race/ethnicity, and rural/urban residence as determined by Rural-Urban Continuum Codes. We also assessed the presence of each of the following comorbid conditions: myocardial infarction, congestive heart failure, peripheral vascular disease, cerebrovascular disease, dementia, chronic pulmonary disease, rheumatologic disease, peptic ulcer disease, mild liver disease, moderate to severe liver disease, diabetes with complications, hemiplegia or paraplegia, renal disease, any

© 2026 Alinezhad et al. *JAMA Network Open*.

malignancy including lymphoma and leukemia (excluding metastatic solid tumors), metastatic solid tumors, and HIV/AIDS.

Detailed Covariate Definitions

Race and ethnicity were obtained from Medicare enrollment information in the Master Beneficiary Summary File and analyzed using the categories available in the data (Asian, Black, Hispanic, North American Native, and White).
To address community context and potential socioeconomic confounding, we included county-level socioeconomic indicators (county population, median household income, poverty rate, unemployment rate, and high school-or-higher educational attainment rate).

To avoid post-baseline covariate leakage , Medicare coverage covariates (months of full Part A/B buy-in and dual eligibility) were computed using monthly indicators in the Master Beneficiary Summary File during the 12 months prior to dialysis initiation.

Follow-up and Censoring Details

Fee-for-service observability was defined monthly based on full Part A and Part B buy-in and no HMO/Medicare Advantage enrollment, using monthly Master Beneficiary Summary File indicators.

Statistical Analysis Details

In constructing time-to-event variables, negative event times (which can occur because of claims timing imprecision) were set to 0, and we added a +1 offset to follow-up time to avoid zero-time issues in the survival TMLE estimation.

Nephrologist–Facility Affiliation and Classification

To identify nephrologists, we used the primary and secondary specialty fields from the Medicare National Downloadable File for each year. If either field included the word “nephrology,” we considered that provider to be a nephrologist. We then gathered information regarding nephrologists’ affiliations with dialysis facilities and these facilities’ characteristics (i.e., ownership status, available dialysis modalities) using Medicare Dialysis Facilities (DFR) data for each year. We matched all outpatient claims with the DFR data using Medicare provider identification numbers and then compiled a list of every dialysis facility from which each nephrologist submitted claims. Our measure of affiliation was determined by linking the outpatient claims and DFR data via matching facility IDs, which identify the facility where each claim was submitted, thereby establishing a connection between the physician’s NPI and the facility. If a nephrologist submitted any claims from a given facility, we deemed them affiliated with that facility. Regarding ownership status, if all claims from a nephrologist came exclusively from for-profit facilities, we classified them as affiliated with for-profit facilities; if they submitted claims from both for-profit and nonprofit facilities, we considered them affiliated with both, and if they only submitted claims from nonprofit facilities, we considered them affiliated with non-profit facilities. The same approach was used for dialysis modalities, attributing to each

© 2026 Alinezhad et al. *JAMA Network Open*.

nephrologist any modalities offered by the facilities from which they submitted claims. Because, on average, each nephrologist submitted claims from between 2 to 3 facilities, restricting them to a single facility would have been imprecise. Consequently, allowing multiple affiliations provided a more accurate representation of each nephrologist's practice environment. Finally, our reference group in all analyses comprised nephrologists who did not submit any claims from any dialysis facilities (i.e., were not affiliated with any facility). It is important to note that being "unaffiliated" does not imply that the nephrologist never sees dialysis patients; rather, it indicates that they do not submit any claims from dedicated dialysis facilities, suggesting that their nephrology care is primarily delivered in alternative settings (e.g., academic medical centers or outpatient clinics). To further characterize the nephrologists in each study group, we assessed both hospital employment and academic practice status. A physician was classified as hospital-employed in a given year if their MD-PPAS practice name included keywords such as "medical center" or "hospital," supplemented by a claims-based approach adapted from Neprash et al. Academic affiliation was determined by identifying practice names containing "university" or "academic."

Practice Setting Characteristics of Not-Affiliated Nephrologists

As anticipated, hospital-employed and academic nephrologists were less common among those affiliated with dialysis facilities compared with those not affiliated (24% vs 48% and 8% vs 18%, respectively).

PD Catheter Timing Restrictions

To ensure PD catheter placement reflected post-dialysis initiation care trajectories, we applied a timing restriction to exclude patients with evidence of PD catheter placement prior to the dialysis initiation baseline, allowing a short pre-baseline window to accommodate planned PD starts and potential claims timing or coding lag (i.e., we excluded PD catheter placements occurring more than 60 days before baseline). This window was chosen to retain patients who initiated dialysis with PD, for whom catheter placement commonly occurs several weeks before dialysis starts. Notably, there were no cases in which a patient's first observed nephrology encounter with their assigned nephrologist occurred after PD catheter placement, reducing concern about reverse temporality.

© 2026 Alinezhad et al. *JAMA Network Open*.

eResults

Study cohort and follow-up

Starting from 38,379 beneficiaries with incident dialysis claims, 28,587 met inclusion criteria after cohort restrictions (excluding non-U.S. states, requiring Medicare eligibility prior to dialysis initiation, excluding ESRD-based Medicare entitlement, and excluding pre-dialysis PD catheter placement, transplant, and death). The primary adjusted analyses included 28,040 beneficiaries with complete baseline covariates.

Average follow-up time was 322 days, reflecting administrative end-of-data truncation for later dialysis initiators and censoring due to loss of fee-for-service observability.

© 2026 Alinezhad et al. *JAMA Network Open*.

eTable 1. Diagnosis Codes and Healthcare Common Procedure Coding System (HCPCS) Codes for Identifying Patients With End-Stage Kidney Disease, Dialysis Procedures, and Kidney Transplantation

Condition	Code Type	Code	Description
End Stage Renal Disease	ICD-10	N186	End stage renal disease
End Stage Renal Disease	ICD-9	5856	End stage renal disease
Dialysis Procedures	ICD-10	Z49	Encounter for care involving renal dialysis
Dialysis Procedures	ICD-10	Z49.0	Preparatory care for renal dialysis
Dialysis Procedures	ICD-10	Z49.01	Encounter for fitting and adjustment of extracorporeal dialysis catheter
Dialysis Procedures	ICD-10	Z49.02	Encounter for fitting and adjustment of peritoneal dialysis catheter
Dialysis Procedures	ICD-10	Z49.3	Encounter for adequacy testing for dialysis
Dialysis Procedures	ICD-10	Z49.31	Encounter for adequacy testing for hemodialysis
Dialysis Procedures	ICD-10	Z49.32	Encounter for adequacy testing for peritoneal dialysis
Dialysis Procedures	ICD-9	V56	Encounter for dialysis
Dialysis Procedures	ICD-9	V561	Encounter for hemodialysis
Dialysis Procedures	ICD-9	V562	Encounter for peritoneal dialysis
Kidney Transplant Status	ICD-10	Z940	Kidney transplant status
Kidney Transplant Status	ICD-9	V420	Kidney transplant status
Kidney Transplant Surgery	ICD-10	0TY	Surgical procedure codes for kidney transplant
Kidney Transplant Surgery	ICD-9	5569	Surgical procedure codes for kidney transplant
Dialysis Services	HCPCS	90951-90970	Dialysis services codes
Dialysis Services	HCPCS	90935	Hemodialysis service with single physician evaluation
Dialysis Services	HCPCS	90937	Hemodialysis service requiring repeated physician evaluations
Dialysis Services	HCPCS	90945	Dialysis procedure for acute kidney failure, without physician evaluation
Dialysis Services	HCPCS	90947	Dialysis procedure for acute kidney failure, requiring repeated evaluations
Peritoneal Dialysis Catheter Placement	CPT	49418	Insertion of tunneled intraperitoneal catheter for dialysis
Peritoneal Dialysis Catheter Placement	CPT	49421	Repositioning or replacement of a tunneled intraperitoneal catheter
Peritoneal Dialysis Catheter Placement	CPT	49324	Laparoscopy, surgical; with insertion of intraperitoneal cannula or catheter
Joint Replacement or Repair Surgery	ICD-10	0SR	Joint replacement procedure codes
Joint Replacement or Repair Surgery	ICD-9	81	Joint replacement procedure codes

© 2026 Alinezhad et al. *JAMA Network Open*.

eFigure 1. Sample Flow Diagram

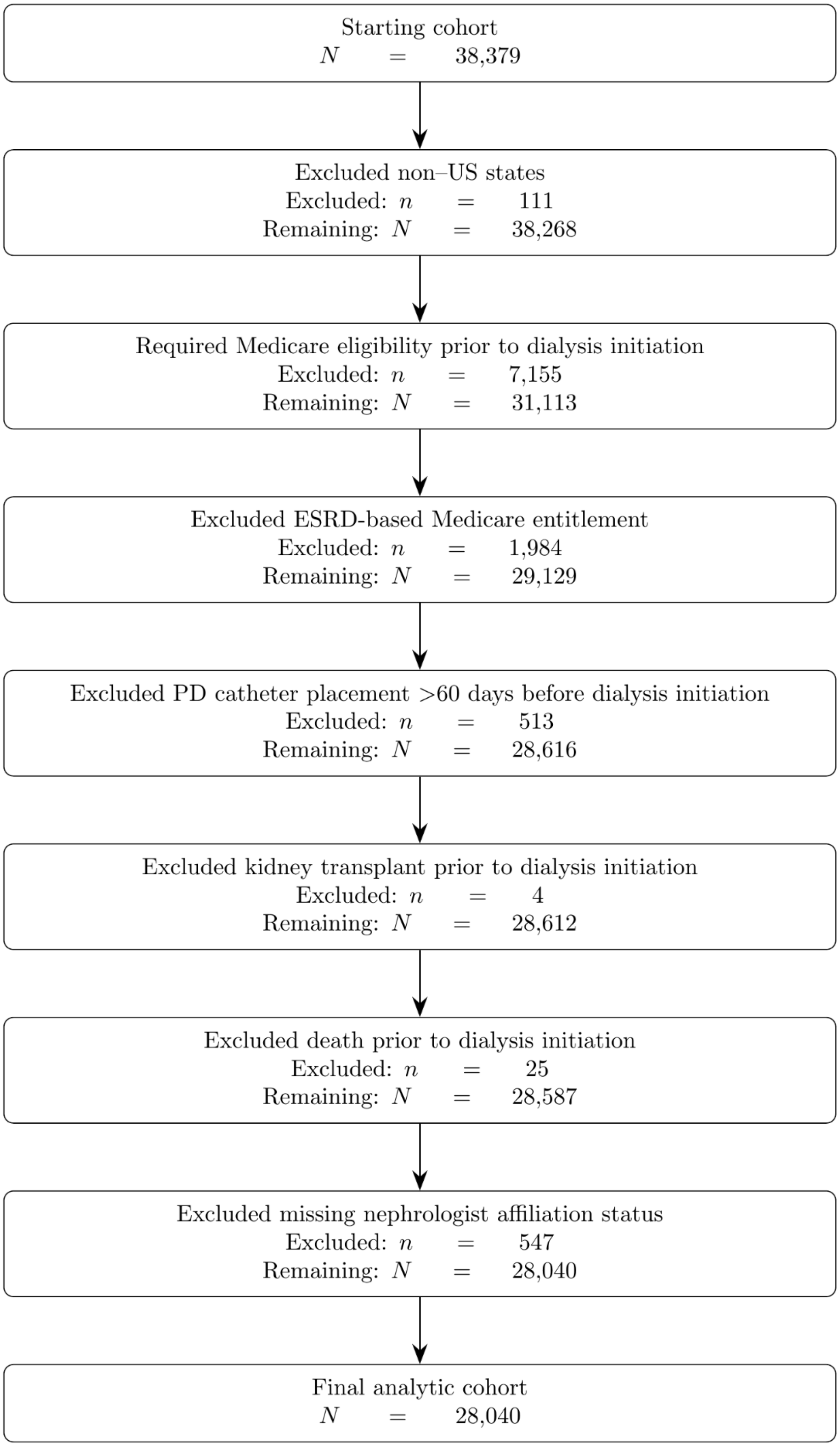

© 2026 Alinezhad et al. *JAMA Network Open*.

eTable 2. Follow-Up Time, Outcome Events, and Censoring in the Analytic Cohort

Metric	Overall	Not affiliated	Affiliated
N	28040	3739	24301
Follow-up, months: Mean (SD)	10.58 (10.08)	9.83 (9.96)	10.76 (10.10)
Death within 24 months, n (%)	4651 (16.30)	682 (18.24)	3865 (15.90)
Transplant within 24 months, n (%)	344 (1.21)	61 (1.63)	279 (1.15)
PD catheter placement within 24 months, n (%)	2304 (8.07)	244 (6.53)	2022 (8.32)
Censored, n (%)	7019 (24.59)	945 (25.27)	5935 (24.42)

Abbreviations: SD, standard deviation

© 2026 Alinezhad et al. *JAMA Network Open*.

eTable 3. Baseline Demographic and Clinical Characteristics of Medicare Beneficiaries Initiating Dialysis

**Characteristic**	**Overall**	**Not affiliated**	**Affiliated**	**SMD**
	N = 28040	N = 3739	N = 24301	
Age, years (Mean (SD))	70.62 (10.83)	69.69 (11.29)	70.76 (10.75)	0.097
Female, %	43.19	43.03	43.22	0.004
Male, %	56.81	56.97	56.78	-0.004
Rural Residence, %	18.76	16.23	19.15	0.076
**Comorbidities, %**				
Myocardial Infarction	24.99	25.49	24.92	-0.013
Congestive Heart Failure	59.69	57.64	60.01	0.048
Peripheral Vascular Disease	36.24	36.05	36.27	0.005
Cerebrovascular Disease	28.85	28.78	28.86	0.002
Dementia	6.92	6.50	6.98	0.019
Chronic Pulmonary Disease	39.46	38.94	39.54	0.012
Rheumatoid Disease	6.03	6.85	5.91	-0.038
Peptic Ulcer Disease	5.02	5.38	4.97	-0.018
Mild Liver Disease	10.86	12.12	10.67	-0.046
Diabetes Mellitus	15.21	15.75	15.13	-0.017
Diabetes with Complications	52.65	48.44	53.29	0.097
Hemiplegia or Paraplegia	3.25	3.69	3.18	-0.028
Cancer (non-metastatic)	16.03	17.41	15.81	-0.043
Moderate to Severe Liver Disease	4.59	7.22	4.18	-0.131
Metastatic Cancer	3.65	4.36	3.54	-0.042
HIV/AIDS	0.81	0.88	0.79	-0.010
Mortality Rate, %	16.22	18.24	15.90	-0.062
Number of Hospitalized Days (Mean (SD))	11.24 (18.78)	12.12 (19.59)	11.10 (18.64)	-0.053
**Race and Ethnicity, %**				
Asian	2.69	2.51	2.71	0.012
Black	21.04	21.32	21.00	-0.008
Hispanic	3.47	3.08	3.53	0.026
North American Native	0.95	1.02	0.94	-0.008
White	68.69	68.76	68.68	-0.002

© 2026 Alinezhad et al. *JAMA Network Open*.

**County-level context, %**				
County population, 100k (Mean (SD))	9.95 (18.40)	11.50 (20.31)	9.71 (18.08)	-0.093
Median household income, $1k (Mean (SD))	59.69 (17.92)	62.25 (18.76)	59.29 (17.75)	-0.162
Poverty rate, % (Mean (SD))	15.00 (5.60)	14.60 (5.61)	15.06 (5.60)	0.081
Unemployment rate, % (Mean (SD))	7.24 (2.71)	7.08 (2.64)	7.26 (2.71)	0.066
High school or higher rate, % (Mean (SD))	49.56 (8.43)	48.33 (8.74)	49.75 (8.37)	0.167
**Outcomes, %**				
Peritoneal Dialysis Catheter Placement	8.08	6.53	8.32	0.068
Kidney Transplant	1.21	1.63	1.15	-0.041
Joint Replacement Surgery	3.00	2.62	3.06	0.027

Abbreviations: HIV/AIDS, human immunodeficiency virus/acquired immunodeficiency syndrome; SD, standard deviation.
[a] Continuous variables are expressed as mean (SD); categorical variables are expressed as percentages.

© 2026 Alinezhad et al. *JAMA Network Open*.

eTable 4. Baseline Demographic and Clinical Characteristics of Medicare Beneficiaries Initiating Dialysis by Nephrologist Affiliation Status and Dialysis Modalities Offered at the Place of Affiliation

**Characteristic**	**Overall**	**Not affiliated**	**Affiliated with both modalities**	**Affiliated with hemodialysis**	**Affiliated with hemodialysis and peritoneal dialysis**
	N = 28021	N = 3756	N = 10372	N = 5286	N = 8607
Age, years (Mean (SD))	70.62 (10.83)	69.69 (11.28)	70.79 (10.69)	70.43 (10.88)	70.93 (10.73)
Female, %	43.20	43.00	43.12	43.95	42.93
Male, %	56.80	57.00	56.88	56.05	57.07
Rural Residence, %	18.77	16.19	21.06	15.25	19.30
**Comorbidities, %**					
Myocardial Infarction	24.97	25.40	24.42	25.20	25.32
Congestive Heart Failure	59.68	57.61	59.32	60.22	60.69
Peripheral Vascular Disease	36.25	36.05	35.44	36.28	37.31
Cerebrovascular Disease	28.85	28.75	28.88	28.55	29.03
Dementia	6.91	6.47	7.03	7.40	6.65
Chronic Pulmonary Disease	39.46	38.90	39.44	40.09	39.33
Rheumatoid Disease	6.04	6.84	5.80	5.90	6.05
Peptic Ulcer Disease	5.02	5.38	4.97	4.71	5.14
Mild Liver Disease	10.86	12.06	9.91	11.52	11.07
Diabetes Mellitus	15.21	15.76	15.07	15.97	14.67
Diabetes with Complications	52.66	48.38	54.16	52.29	52.95
Hemiplegia or Paraplegia	3.24	3.70	3.32	3.01	3.10
Cancer (non-metastatic)	16.03	17.44	15.13	14.98	17.15
Moderate to Severe Liver Disease	4.59	7.19	3.97	4.26	4.40
Metastatic Cancer	3.64	4.37	3.19	3.22	4.14
HIV/AIDS	0.80	0.88	0.67	1.12	0.74
Mortality Rate, %	16.22	18.21	15.40	15.93	16.52
Number of Hospitalized Days (Mean (SD))	11.24 (18.78)	12.11 (19.61)	10.76 (18.10)	11.14 (19.28)	11.49 (18.88)
**Race and Ethnicity, %**					
Asian	2.69	2.53	2.65	3.08	2.56

© 2026 Alinezhad et al. *JAMA Network Open*.

Black	21.03	21.27	21.68	23.25	18.78
Hispanic	3.48	3.06	3.87	3.93	2.90
North American Native	0.95	1.01	0.99	1.04	0.82
White	68.70	68.80	67.66	65.19	72.06
**County-level context, %**					
County population, 100k (Mean (SD))	9.95 (18.39)	11.51 (20.30)	8.50 (16.87)	12.50 (19.86)	9.45 (18.12)
Median household income, $1k (Mean (SD))	59.69 (17.92)	62.28 (18.76)	57.70 (17.54)	60.11 (17.68)	60.70 (17.89)
Poverty rate, % (Mean (SD))	15.00 (5.60)	14.59 (5.61)	15.58 (5.80)	15.19 (5.43)	14.35 (5.36)
Unemployment rate, % (Mean (SD))	7.24 (2.71)	7.08 (2.64)	7.32 (2.79)	7.41 (2.75)	7.09 (2.60)
High school or higher rate, % (Mean (SD))	49.56 (8.43)	48.32 (8.74)	50.37 (8.25)	48.69 (8.23)	49.68 (8.53)
**Outcomes, %**					
Peritoneal Dialysis Catheter Placement	8.08	6.50	8.91	6.70	8.62
Kidney Transplant	1.21	1.62	1.21	1.08	1.13
Joint Replacement Surgery	3.00	2.61	2.96	3.18	3.10

Abbreviations: HIV/AIDS, human immunodeficiency virus/acquired immunodeficiency syndrome; SD, standard deviation.
[a] Continuous variables are expressed as mean (SD); categorical variables are expressed as percentages.
[b] The difference in the total number of cases between this table and Table 1 arises from additional missing data on nephrologist modality stat

© 2026 Alinezhad et al. *JAMA Network Open*.

eFigure 2. Adjusted Differences in Peritoneal Dialysis Catheter Placement, Kidney Transplant Surgery, and Joint Replacement Surgery by Nephrologist Affiliation, Profit Status, and Dialysis Modalities Offered (Strong Affiliation ≥75%)

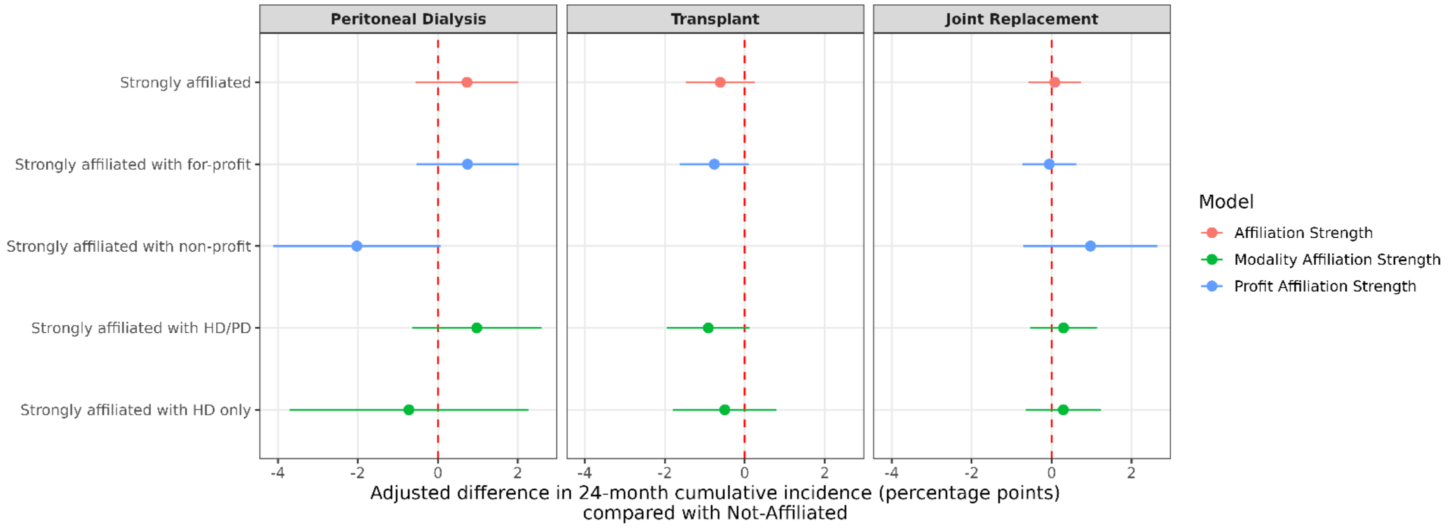

Outcome	Comparison	Risk difference (pp)	95% CI	Holm p-value	BH p-value
Peritoneal Dialysis	Strongly affiliated	0.73	(-0.56, 2.02)	0.96	0.34
Peritoneal Dialysis	Strongly affiliated with for-profit	0.74	(-0.54, 2.02)	0.96	0.34
Peritoneal Dialysis	Strongly affiliated with non-profit	-2.03	(-4.12, 0.07)	0.29	0.29
Peritoneal Dialysis	Strongly affiliated with HD/PD	0.97	(-0.65, 2.59)	0.96	0.34
Peritoneal Dialysis	Strongly affiliated with HD only	-0.72	(-3.71, 2.26)	0.96	0.63
Transplant	Strongly affiliated	-0.61	(-1.48, 0.25)	0.33	0.22
Transplant	Strongly affiliated with for-profit	-0.76	(-1.62, 0.10)	0.33	0.17
Transplant	Strongly affiliated with non-profit	–	–	–	–
Transplant	Strongly affiliated with HD/PD	-0.91	(-1.95, 0.13)	0.33	0.17
Transplant	Strongly affiliated with HD only	-0.50	(-1.79, 0.80)	0.45	0.45
Joint Replacement	Strongly affiliated	0.08	(-0.58, 0.74)	1.00	0.87
Joint Replacement	Strongly affiliated with for-profit	-0.06	(-0.73, 0.62)	1.00	0.87
Joint Replacement	Strongly affiliated with non-profit	0.97	(-0.70, 2.65)	1.00	0.87
Joint Replacement	Strongly affiliated with HD/PD	0.30	(-0.54, 1.14)	1.00	0.87
Joint Replacement	Strongly affiliated with HD only	0.29	(-0.64, 1.23)	1.00	0.87

Abbreviations: HD, hemodialysis; HD/PD, hemodialysis and peritoneal dialysis.
Points show adjusted 24-month risk differences (percentage points) compared with the Not Affiliated reference group; horizontal lines indicate 95% CIs. Estimates were obtained from separate doubly robust survival targeted maximum likelihood estimation (TMLE) models for (1) affiliation overall, (2) affiliation stratified by dialysis-facility profit status, and (3) affiliation stratified by dialysis modalities offered, restricting the sample to nephrologists with strong affiliation patterns (≥75% of outpatient claims consistent with the assigned category). For PD catheter placement, death and kidney transplant were treated as competing events; for transplant and joint replacement surgery, death was treated as a competing event. The red dashed vertical line indicates no difference (risk difference = 0). The accompanying table reports point estimates, 95% CIs, and Holm and Benjamini–Hochberg multiplicity-adjusted P values within each outcome. In some subgroup and sensitivity analyses, estimates are not shown because the corresponding models did not converge, reflecting limited effective sample size and/or a small number of outcome events in those strata.

© 2026 Alinezhad et al. *JAMA Network Open*.

eFigure 3. Adjusted Differences in Peritoneal Dialysis Catheter Placement, Kidney Transplant Surgery, and Joint Replacement Surgery by Nephrologist Affiliation, Profit Status, and Dialysis Modalities Offered (Dialysis Initiation Through 2019; Pre–COVID-19 Era)

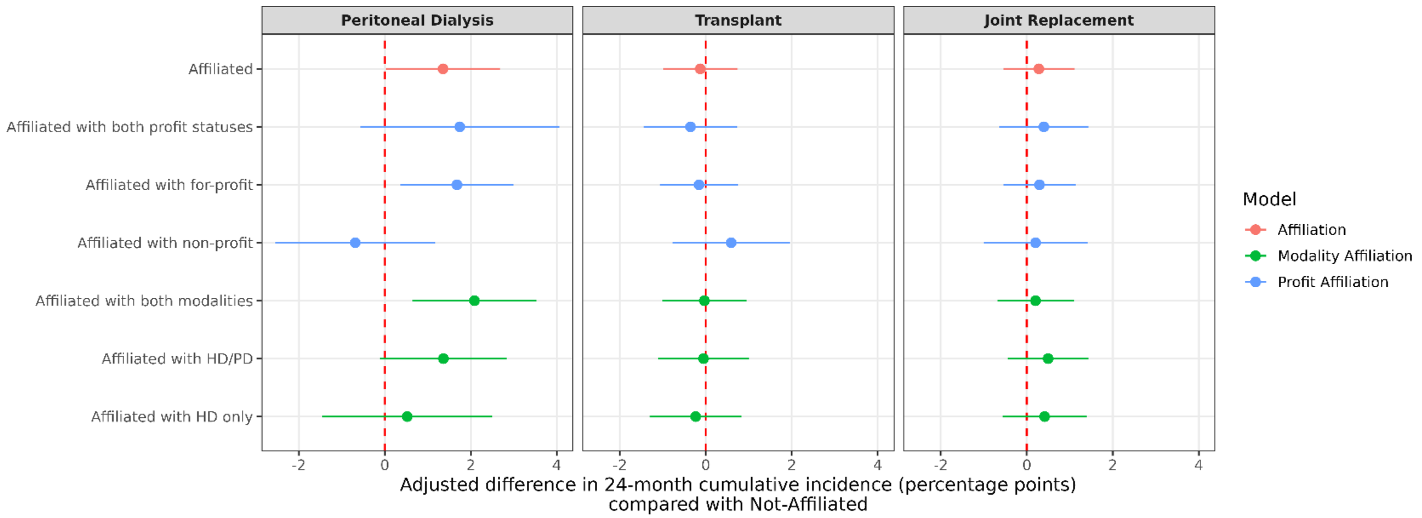

Outcome	Comparison	Risk difference (pp)	95% CI	Holm p-value	BH p-value
Peritoneal Dialysis	Affiliated	1.36	(0.03, 2.68)	0.23	0.11
Peritoneal Dialysis	Affiliated with both profit statuses	1.74	(-0.57, 4.06)	0.42	0.20
Peritoneal Dialysis	Affiliated with for-profit	1.68	(0.36, 3.00)	0.08	0.04
Peritoneal Dialysis	Affiliated with non-profit	-0.69	(-2.54, 1.17)	0.94	0.55
Peritoneal Dialysis	Affiliated with both modalities	2.09	(0.65, 3.53)	0.03	0.03
Peritoneal Dialysis	Affiliated with HD/PD	1.36	(-0.11, 2.84)	0.28	0.12
Peritoneal Dialysis	Affiliated with HD only	0.52	(-1.45, 2.50)	0.94	0.60
Transplant	Affiliated	-0.13	(-1.00, 0.74)	1.00	0.95
Transplant	Affiliated with both profit statuses	-0.35	(-1.44, 0.73)	1.00	0.95
Transplant	Affiliated with for-profit	-0.16	(-1.06, 0.75)	1.00	0.95
Transplant	Affiliated with non-profit	0.59	(-0.78, 1.96)	1.00	0.95
Transplant	Affiliated with both modalities	-0.03	(-1.01, 0.95)	1.00	0.95
Transplant	Affiliated with HD/PD	-0.05	(-1.11, 1.00)	1.00	0.95
Transplant	Affiliated with HD only	-0.24	(-1.30, 0.83)	1.00	0.95
Joint Replacement	Affiliated	0.28	(-0.55, 1.11)	1.00	0.71
Joint Replacement	Affiliated with both profit statuses	0.40	(-0.64, 1.43)	1.00	0.71
Joint Replacement	Affiliated with for-profit	0.29	(-0.54, 1.13)	1.00	0.71
Joint Replacement	Affiliated with non-profit	0.20	(-1.01, 1.41)	1.00	0.74
Joint Replacement	Affiliated with both modalities	0.20	(-0.69, 1.09)	1.00	0.74
Joint Replacement	Affiliated with HD/PD	0.50	(-0.44, 1.43)	1.00	0.71

Abbreviations: HD, hemodialysis; HD/PD, hemodialysis and peritoneal dialysis.
Points show adjusted 24-month risk differences (percentage points) compared with the Not Affiliated reference group; horizontal lines indicate 95% CIs. Estimates were obtained from separate doubly robust survival targeted maximum likelihood estimation (TMLE) models for (1) affiliation overall, (2) affiliation stratified by dialysis-facility profit status, and (3) affiliation stratified by dialysis modalities offered, restricting the cohort to patients initiating dialysis through 2019. For PD catheter placement, death and kidney transplant were treated as competing events; for transplant and joint replacement surgery, death was treated as a competing event. The red dashed vertical line indicates no difference (risk difference = 0). The accompanying table reports point estimates, 95% CIs, and Holm and Benjamini–Hochberg multiplicity-adjusted P values within each outcome.

© 2026 Alinezhad et al. *JAMA Network Open*.

eFigure 4. Adjusted Differences in Peritoneal Dialysis Catheter Placement, Kidney Transplant Surgery, and Joint Replacement Surgery by Nephrologist Affiliation, Profit Status, and Dialysis Modalities Offered (No Crash Starts)

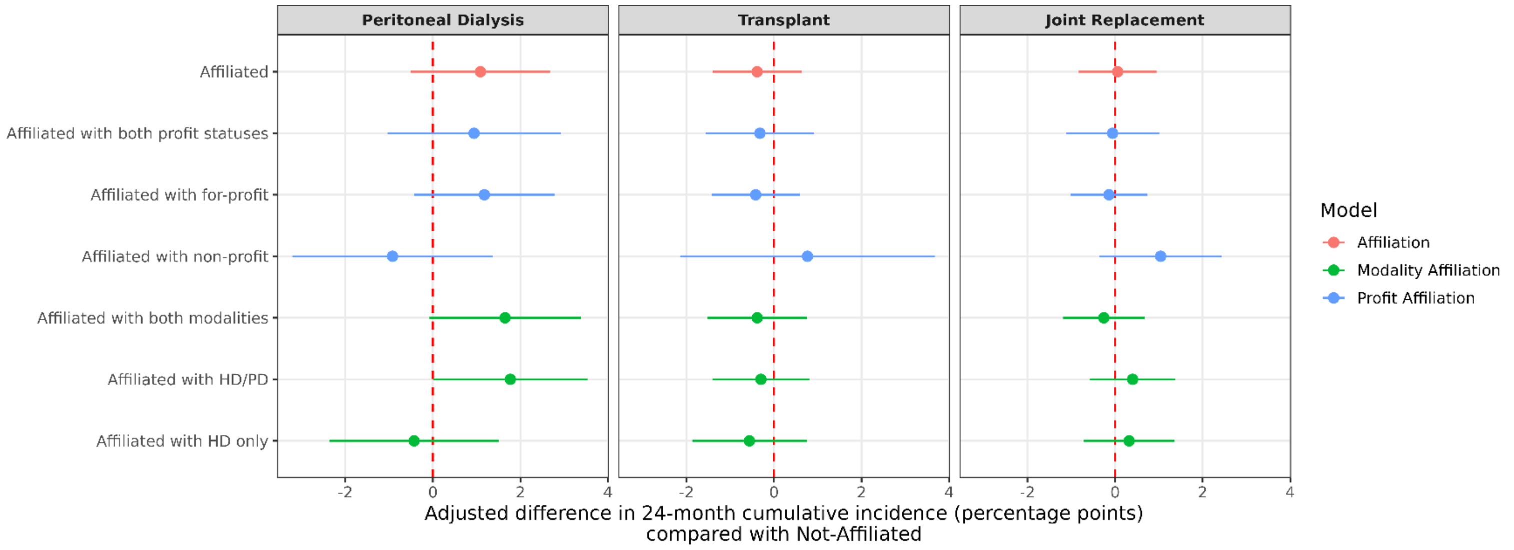

Outcome	Comparison	Risk difference (pp)	95% CI	Holm p-value	BH p-value
Peritoneal Dialysis	Affiliated	1.09	(-0.50, 2.67)	0.76	0.32
Peritoneal Dialysis	Affiliated with both profit statuses	0.94	(-1.03, 2.92)	1.00	0.49
Peritoneal Dialysis	Affiliated with for-profit	1.18	(-0.43, 2.78)	0.76	0.32
Peritoneal Dialysis	Affiliated with non-profit	-0.92	(-3.21, 1.36)	1.00	0.50
Peritoneal Dialysis	Affiliated with both modalities	1.65	(-0.08, 3.38)	0.37	0.21
Peritoneal Dialysis	Affiliated with HD/PD	1.77	(0.01, 3.53)	0.34	0.21
Peritoneal Dialysis	Affiliated with HD only	-0.43	(-2.36, 1.50)	1.00	0.66
Transplant	Affiliated	-0.38	(-1.40, 0.63)	1.00	0.61
Transplant	Affiliated with both profit statuses	-0.32	(-1.56, 0.91)	1.00	0.61
Transplant	Affiliated with for-profit	-0.42	(-1.42, 0.59)	1.00	0.61
Transplant	Affiliated with non-profit	0.77	(-2.14, 3.67)	1.00	0.61
Transplant	Affiliated with both modalities	-0.38	(-1.52, 0.76)	1.00	0.61
Transplant	Affiliated with HD/PD	-0.30	(-1.41, 0.81)	1.00	0.61
Transplant	Affiliated with HD only	-0.56	(-1.86, 0.75)	1.00	0.61
Joint Replacement	Affiliated	0.06	(-0.83, 0.95)	1.00	0.92
Joint Replacement	Affiliated with both profit statuses	-0.06	(-1.12, 1.01)	1.00	0.92
Joint Replacement	Affiliated with for-profit	-0.14	(-1.02, 0.74)	1.00	0.92
Joint Replacement	Affiliated with non-profit	1.04	(-0.36, 2.44)	1.00	0.92
Joint Replacement	Affiliated with both modalities	-0.25	(-1.19, 0.68)	1.00	0.92
Joint Replacement	Affiliated with HD/PD	0.40	(-0.57, 1.38)	1.00	0.92

Abbreviations: HD, hemodialysis; HD/PD, hemodialysis and peritoneal dialysis.
Points show adjusted 24-month risk differences (percentage points) compared with the Not Affiliated reference group; horizontal lines indicate 95% CIs. Estimates were obtained from separate doubly robust survival targeted maximum likelihood estimation (TMLE) models for (1) affiliation overall, (2) affiliation stratified by dialysis-facility profit status, and (3) affiliation stratified by dialysis modalities offered, restricting the cohort to patients whose first observed nephrology encounter occurred at least 30 days before dialysis initiation ("no crash starts"). For PD catheter placement, death and kidney transplant were treated as competing events; for transplant and joint replacement surgery, death was treated as a competing event. The red dashed vertical line indicates no difference (risk difference = 0). The accompanying table reports point estimates, 95% CIs, and Holm and Benjamini–Hochberg multiplicity-adjusted P values within each outcome.

© 2026 Alinezhad et al. *JAMA Network Open*.

eFigure 5. Sex-Stratified Adjusted Differences in Peritoneal Dialysis Catheter Placement by Nephrologist Affiliation, Profit Status, and Dialysis Modalities Offered

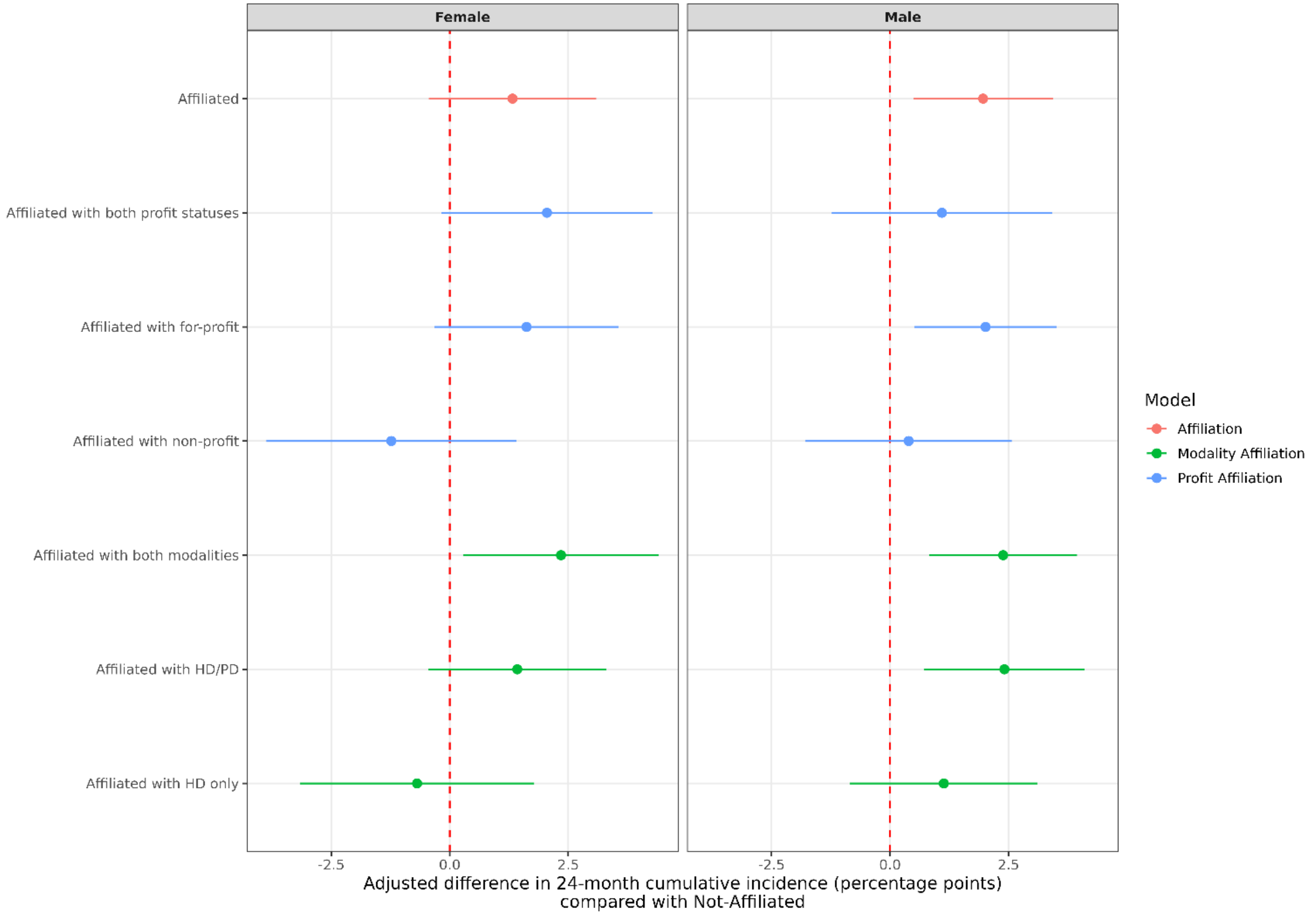

Abbreviations: HD, hemodialysis; HD/PD, hemodialysis and peritoneal dialysis.
Points show adjusted 24-month risk differences (percentage points) compared with the Not Affiliated reference group; horizontal lines indicate 95% CIs. Estimates were obtained from separate doubly robust survival targeted maximum likelihood estimation (TMLE) models fit within male and female subgroups. For PD catheter placement, death and kidney transplant were treated as competing events. The red dashed vertical line indicates no difference (risk difference = 0).

© 2026 Alinezhad et al. *JAMA Network Open*.

eFigure 6. Race and Ethnicity–Stratified Adjusted Differences in Peritoneal Dialysis Catheter Placement by Nephrologist Affiliation, Profit Status, and Dialysis Modalities Offered

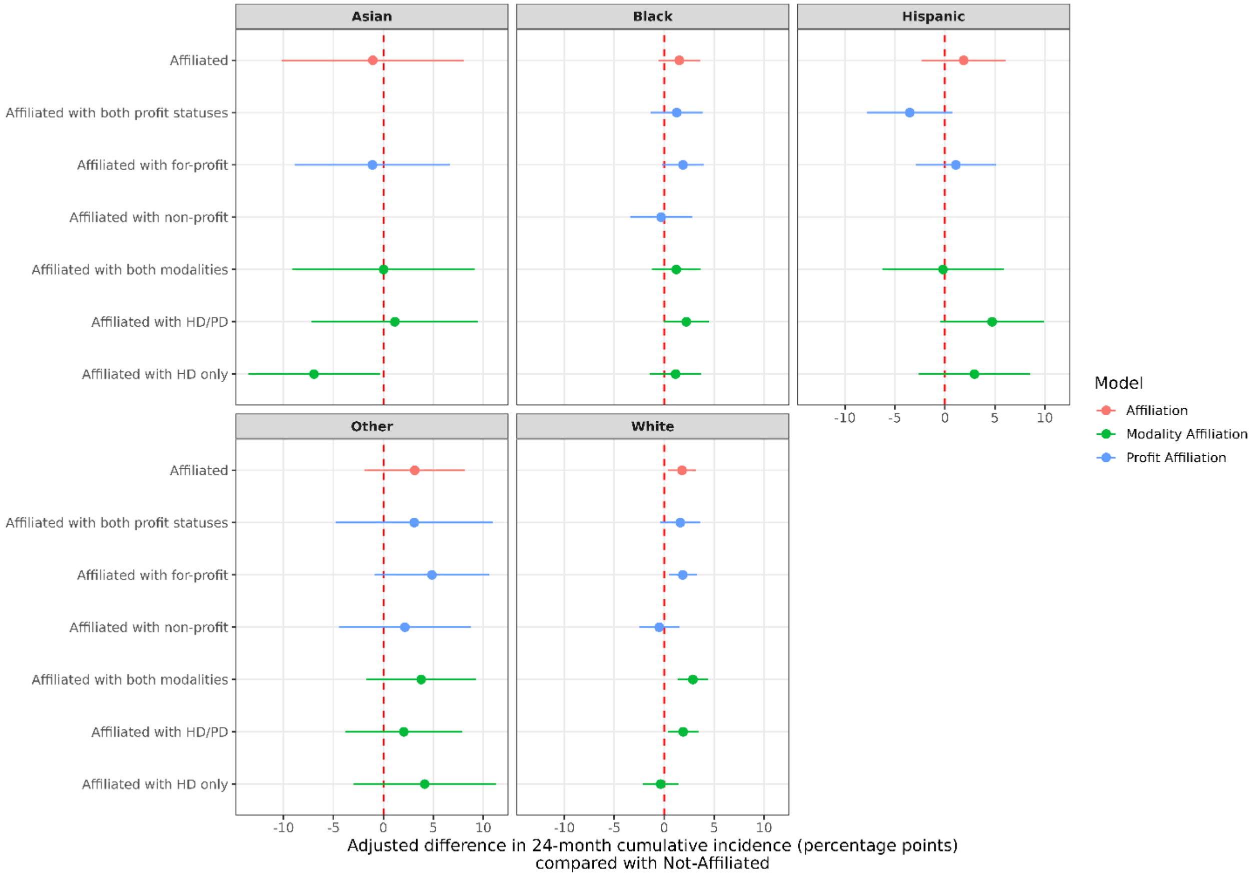

Abbreviations: HD, hemodialysis; HD/PD, hemodialysis and peritoneal dialysis.
Points show adjusted 24-month risk differences (percentage points) compared with the Not Affiliated reference group; horizontal lines indicate 95% CIs. Estimates were obtained from separate doubly robust survival targeted maximum likelihood estimation (TMLE) models fit within race and ethnicity subgroups. For PD catheter placement, death and kidney transplant were treated as competing events. The red dashed vertical line indicates no difference (risk difference = 0). In some subgroup and sensitivity analyses, estimates are not shown because the corresponding models did not converge, reflecting limited effective sample size and/or a small number of outcome events in those strata.

© 2026 Alinezhad et al. *JAMA Network Open*.

eFigure 7. Rurality-Stratified Adjusted Differences in Peritoneal Dialysis Catheter Placement by Nephrologist Affiliation, Profit Status, and Dialysis Modalities Offered

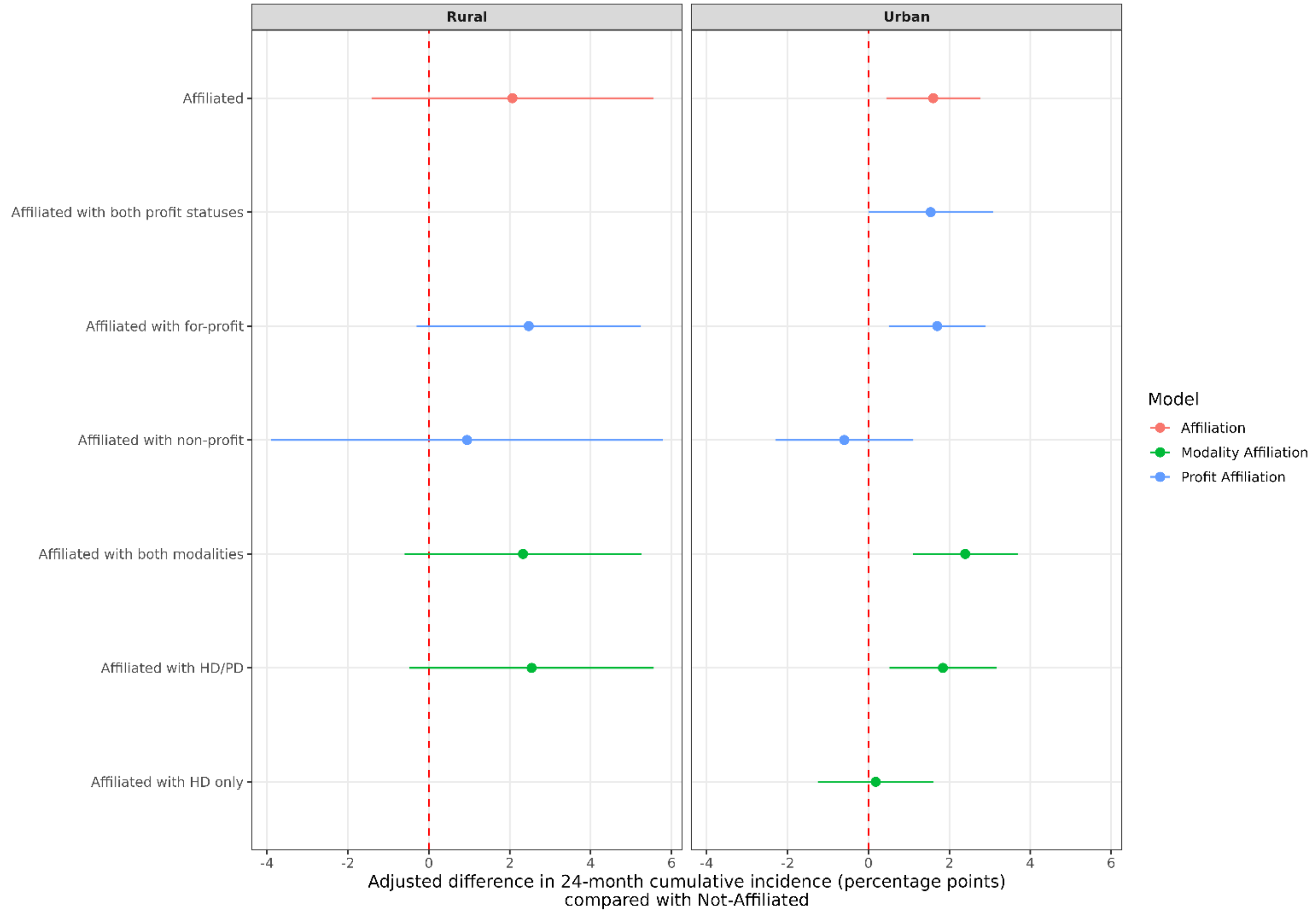

Abbreviations: HD, hemodialysis; HD/PD, hemodialysis and peritoneal dialysis.
Points show adjusted 24-month risk differences (percentage points) compared with the Not Affiliated reference group; horizontal lines indicate 95% CIs. Estimates were obtained from separate doubly robust survival targeted maximum likelihood estimation (TMLE) models fit within urban and rural subgroups. For PD catheter placement, death and kidney transplant were treated as competing events. The red dashed vertical line indicates no difference (risk difference = 0). In some subgroup and sensitivity analyses, estimates are not shown because the corresponding models did not converge, reflecting limited effective sample size and/or a small number of outcome events in those strata.

© 2026 Alinezhad et al. *JAMA Network Open*.
