# Supplementary material for: Nephrologist Affiliation With Dialysis Facilities and Patient Trajectories in End-Stage Kidney Disease
Source: JAMA Netw Open. 2026 Apr 16;9(4):e266156. doi: 10.1001/jamanetworkopen.2026.6156 (PMC13087812; doi:10.1001/jamanetworkopen.2026.6156)
Supplement: Supplement 2. — Data Sharing Statement [file jamanetwopen-e266156-s002.pdf]

Data Sharing Statement

Alinezhad. Nephrologist Affiliation With Dialysis Facilities and Patient Trajectories in End-Stage Kidney Disease. *JAMA Netw Open*. Published April 16, 2026.
doi:10.1001/jamanetworkopen.2026.6156

Data
Data available: No

Additional Information
Explanation for why data not available: Data use agreement and patient privacy
